# 2-Benzoyl-1*H*-benzimidazole

**DOI:** 10.1107/S1600536810045666

**Published:** 2010-11-13

**Authors:** Lin Ai, Xiu-Min Shen, Seik Weng Ng

**Affiliations:** a College of Chemistry, Beijing Normal University, Beijing 100875, People’s Republic of China; b Department of Chemistry, University of Malaya, 50603 Kuala Lumpur, Malaysia

## Abstract

In the title compound, C_14_H_10_N_2_O, the benzoyl ring and benzimidazole ring system are aligned at a dihedral angle of 50.2 (2)°. In the crystal, inter­molecular N—H⋯N hydrogen bonds between adjacent imidazole groups generate supra­molecular *C*(4) chains running along the *b* axis.

## Related literature

For phototropism of 2-acetyl­benzimidazole and 2-benzoyl­benzimidazole, see: Chowdhury *et al.* (2005 ▶). For the crystal structure of 2-acetyl­benzimidazole, see: Yang *et al.* (2006 ▶).
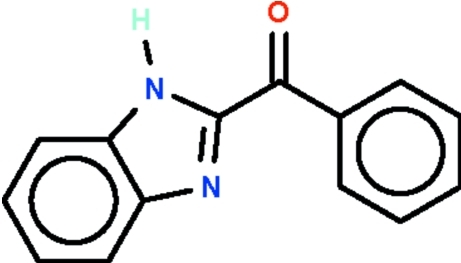


## Experimental

### 

#### Crystal data

C_14_H_10_N_2_O
*M* *_r_* = 222.24
Orthorhombic, 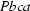

*a* = 14.7356 (8) Å
*b* = 9.9530 (12) Å
*c* = 15.7981 (12) Å
*V* = 2317.0 (4) Å^3^
*Z* = 8
Mo *K*α radiation
μ = 0.08 mm^−1^
*T* = 293 K
0.40 × 0.40 × 0.20 mm

#### Data collection

Bruker SMART APEX diffractometer
10324 measured reflections
2658 independent reflections
1885 reflections with *I* > 2σ(*I*)
*R*_int_ = 0.030

#### Refinement

*R*[*F* ^2^ > 2σ(*F* ^2^)] = 0.039
*wR*(*F* ^2^) = 0.115
*S* = 1.01
2658 reflections
159 parameters
H atoms treated by a mixture of independent and constrained refinement
Δρ_max_ = 0.16 e Å^−3^
Δρ_min_ = −0.14 e Å^−3^

### 

Data collection: *APEX2* (Bruker, 2007 ▶); cell refinement: *SAINT* (Bruker, 2007 ▶); data reduction: *SAINT*; program(s) used to solve structure: *SHELXS97* (Sheldrick, 2008 ▶); program(s) used to refine structure: *SHELXL97* (Sheldrick, 2008 ▶); molecular graphics: *X-SEED* (Barbour, 2001 ▶); software used to prepare material for publication: *publCIF* (Westrip, 2010 ▶).

## Supplementary Material

Crystal structure: contains datablocks global, I. DOI: 10.1107/S1600536810045666/xu5084sup1.cif

Structure factors: contains datablocks I. DOI: 10.1107/S1600536810045666/xu5084Isup2.hkl

Additional supplementary materials:  crystallographic information; 3D view; checkCIF report

## Figures and Tables

**Table 1:** Hydrogen-bond geometry (Å, °)

*D*—H⋯*A*	*D*—H	H⋯*A*	*D*⋯*A*	*D*—H⋯*A*
N1—H1⋯N2^i^	0.90 (2)	1.95 (2)	2.829 (2)	164 (1)

Symmetry code: (i) 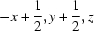.

**Fig. 1.**
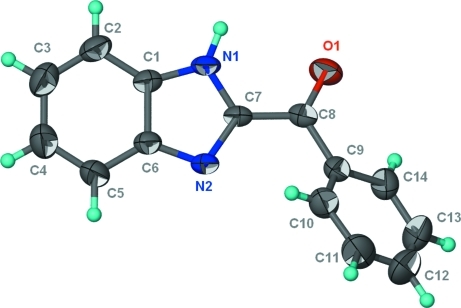
Thermal ellipsoid plot (Barbour, 2001) of C14H11N2O at the 50% probability level; hydrogen atoms are drawn as spheres of arbitrary radius.

**Fig. 2.**
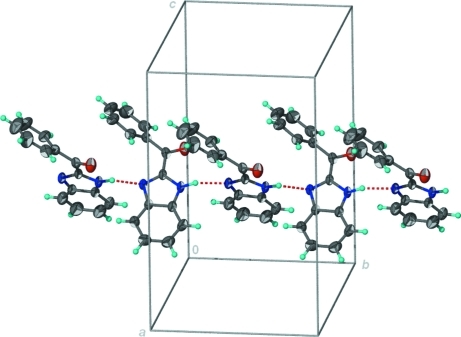
Hydrogen-bonded chain structure.

## supplementary crystallographic information

### Comment

Acetyl-2-benzimidazole and benzoyl-2-benzimidazole are reported to exhibit excited-state prototropism in solvents at different pH levels (Chowdhury *et al.*, 2005). Acetyl-2-benzimidazole exists in the solid state as an N–H···O hydrogen bonded dimer; the molecule is essentially planar (Yang *et al.*, 2006). With the larger phenyl ring in place of the methyl group, the aromatic analog (Scheme I) requires rotation of the aromatic ring in order to reduce strain; this is reflected in the 50.2 (2) ° dihedral angle between the phenyl and benzimidazolyl rings (Fig. 1). Adjacent molecules are linked into a chan by N–H···O hydrogen bonds (Fig. 2).

### Experimental

The compound was synthesized by using a literature procedure (Chowdhury *et al.*, 2005), and crystals were grown from a methanol solution of the compound.

### Refinement

Carbon-bound H-atoms were placed in calculated positions (C–H 0.93 Å) and were included in the refinement in the riding model approximation, with *U*_iso_(H) set to 1.2*U*_eq_(C).

The amino H-atom was located in a difference Fourier map and was refined isotropically.

### Crystal data

**Table:**

C_14_H_10_N_2_O	*F*(000) = 928
*M**_r_* = 222.24	*D*_x_ = 1.274 Mg m^−^^3^
Orthorhombic, *P**b**c**a*	Mo *K*α radiation, λ = 0.71073 Å
Hall symbol: -P 2ac 2ab	Cell parameters from 2496 reflections
*a* = 14.7356 (8) Å	θ = 2.8–26.7°
*b* = 9.9530 (12) Å	µ = 0.08 mm^−^^1^
*c* = 15.7981 (12) Å	*T* = 293 K
*V* = 2317.0 (4) Å^3^	Block, colorless
*Z* = 8	0.40 × 0.40 × 0.20 mm

### Data collection

**Table:**

Bruker SMART APEX diffractometer	1885 reflections with *I* > 2σ(*I*)
Radiation source: fine-focus sealed tube	*R*_int_ = 0.030
graphite	θ_max_ = 27.5°, θ_min_ = 2.8°
ω scans	*h* = −10→19
10324 measured reflections	*k* = −12→11
2658 independent reflections	*l* = −15→20

### Refinement

**Table:**

Refinement on *F*^2^	Secondary atom site location: difference Fourier map
Least-squares matrix: full	Hydrogen site location: inferred from neighbouring sites
*R*[*F*^2^ > 2σ(*F*^2^)] = 0.039	H atoms treated by a mixture of independent and constrained refinement
*wR*(*F*^2^) = 0.115	*w* = 1/[σ^2^(*F*_o_^2^) + (0.0549*P*)^2^ + 0.2609*P*] where *P* = (*F*_o_^2^ + 2*F*_c_^2^)/3
*S* = 1.01	(Δ/σ)_max_ = 0.001
2658 reflections	Δρ_max_ = 0.16 e Å^−^^3^
159 parameters	Δρ_min_ = −0.13 e Å^−^^3^
0 restraints	Extinction correction: *SHELXL97* (Sheldrick, 2008), Fc^*^=kFc[1+0.001xFc^2^λ^3^/sin(2θ)]^-1/4^
Primary atom site location: structure-invariant direct methods	Extinction coefficient: 0.0093 (12)

### Fractional atomic coordinates and isotropic or equivalent isotropic displacement parameters (Å^2^)

**Table:**

	*x*	*y*	*z*	*U*_iso_*/*U*_eq_	
O1	0.38338 (7)	0.51103 (10)	0.45822 (7)	0.0647 (3)	
N1	0.23286 (7)	0.52044 (10)	0.35142 (7)	0.0456 (3)	
H1	0.2500 (10)	0.6048 (17)	0.3643 (9)	0.066 (4)*	
N2	0.22405 (7)	0.29711 (9)	0.35955 (7)	0.0450 (3)	
C1	0.16927 (9)	0.48272 (12)	0.29302 (8)	0.0446 (3)	
C2	0.11547 (10)	0.55500 (15)	0.23652 (9)	0.0609 (4)	
H2	0.1189	0.6481	0.2328	0.073*	
C3	0.05747 (12)	0.48239 (18)	0.18687 (10)	0.0733 (5)	
H3	0.0207	0.5274	0.1483	0.088*	
C4	0.05192 (13)	0.34275 (18)	0.19233 (10)	0.0767 (5)	
H4	0.0111	0.2972	0.1577	0.092*	
C5	0.10482 (11)	0.27110 (15)	0.24714 (10)	0.0647 (4)	
H5	0.1011	0.1779	0.2501	0.078*	
C6	0.16460 (9)	0.34273 (12)	0.29846 (8)	0.0453 (3)	
C7	0.26312 (9)	0.40660 (11)	0.38936 (7)	0.0411 (3)	
C8	0.33717 (9)	0.41037 (12)	0.45261 (8)	0.0444 (3)	
C9	0.35378 (9)	0.28972 (13)	0.50523 (8)	0.0471 (3)	
C10	0.28375 (11)	0.21174 (15)	0.53695 (9)	0.0613 (4)	
H10	0.2238	0.2326	0.5240	0.074*	
C11	0.30339 (17)	0.10285 (19)	0.58787 (11)	0.0904 (7)	
H11	0.2566	0.0520	0.6110	0.108*	
C12	0.3922 (2)	0.0697 (2)	0.60441 (13)	0.1081 (8)	
H12	0.4052	−0.0053	0.6373	0.130*	
C13	0.46157 (16)	0.1462 (2)	0.57286 (13)	0.0942 (7)	
H13	0.5214	0.1224	0.5840	0.113*	
C14	0.44330 (11)	0.25789 (16)	0.52484 (9)	0.0632 (4)	
H14	0.4904	0.3119	0.5055	0.076*	

### Atomic displacement parameters (Å^2^)

**Table:**

	*U*^11^	*U*^22^	*U*^33^	*U*^12^	*U*^13^	*U*^23^
O1	0.0593 (6)	0.0444 (6)	0.0904 (8)	−0.0143 (5)	−0.0074 (5)	0.0005 (5)
N1	0.0576 (7)	0.0243 (5)	0.0551 (6)	−0.0016 (5)	0.0001 (5)	0.0018 (5)
N2	0.0574 (7)	0.0269 (5)	0.0506 (6)	−0.0008 (4)	−0.0037 (5)	0.0015 (4)
C1	0.0526 (7)	0.0335 (6)	0.0477 (7)	0.0013 (5)	0.0040 (6)	0.0032 (5)
C2	0.0731 (10)	0.0441 (8)	0.0656 (9)	0.0079 (7)	−0.0038 (8)	0.0136 (7)
C3	0.0784 (11)	0.0751 (11)	0.0663 (9)	0.0043 (9)	−0.0174 (9)	0.0174 (9)
C4	0.0894 (13)	0.0752 (12)	0.0656 (9)	−0.0134 (10)	−0.0262 (9)	0.0048 (9)
C5	0.0858 (11)	0.0455 (8)	0.0630 (8)	−0.0114 (7)	−0.0168 (8)	0.0004 (7)
C6	0.0561 (8)	0.0329 (6)	0.0469 (6)	−0.0008 (5)	−0.0013 (6)	0.0019 (5)
C7	0.0486 (7)	0.0273 (6)	0.0475 (6)	−0.0014 (5)	0.0044 (5)	0.0009 (5)
C8	0.0437 (7)	0.0354 (6)	0.0543 (7)	−0.0020 (5)	0.0046 (6)	−0.0045 (6)
C9	0.0559 (8)	0.0403 (7)	0.0450 (6)	−0.0009 (6)	−0.0049 (6)	−0.0037 (6)
C10	0.0725 (10)	0.0583 (9)	0.0531 (8)	−0.0141 (7)	−0.0062 (7)	0.0077 (7)
C11	0.1331 (19)	0.0743 (12)	0.0637 (10)	−0.0338 (12)	−0.0211 (11)	0.0238 (9)
C12	0.160 (2)	0.0778 (14)	0.0864 (13)	−0.0021 (15)	−0.0520 (15)	0.0288 (11)
C13	0.1035 (16)	0.0826 (14)	0.0967 (14)	0.0211 (12)	−0.0447 (12)	0.0051 (12)
C14	0.0632 (9)	0.0625 (9)	0.0641 (9)	0.0056 (7)	−0.0151 (7)	−0.0065 (7)

### Geometric parameters (Å, °)

**Table:**

O1—C8	1.2146 (14)	C5—H5	0.9300
N1—C7	1.3571 (15)	C7—C8	1.4800 (18)
N1—C1	1.3677 (17)	C8—C9	1.4809 (18)
N1—H1	0.900 (17)	C9—C10	1.3851 (19)
N2—C7	1.3195 (15)	C9—C14	1.392 (2)
N2—C6	1.3802 (16)	C10—C11	1.380 (2)
C1—C2	1.3938 (19)	C10—H10	0.9300
C1—C6	1.3977 (17)	C11—C12	1.375 (3)
C2—C3	1.367 (2)	C11—H11	0.9300
C2—H2	0.9300	C12—C13	1.369 (3)
C3—C4	1.395 (2)	C12—H12	0.9300
C3—H3	0.9300	C13—C14	1.372 (2)
C4—C5	1.366 (2)	C13—H13	0.9300
C4—H4	0.9300	C14—H14	0.9300
C5—C6	1.3933 (19)		
			
C7—N1—C1	107.09 (10)	N2—C7—C8	125.70 (11)
C7—N1—H1	125.9 (10)	N1—C7—C8	121.28 (10)
C1—N1—H1	127.0 (10)	O1—C8—C7	118.92 (12)
C7—N2—C6	104.76 (10)	O1—C8—C9	122.35 (12)
N1—C1—C2	132.85 (12)	C7—C8—C9	118.71 (10)
N1—C1—C6	105.42 (11)	C10—C9—C14	119.88 (13)
C2—C1—C6	121.73 (13)	C10—C9—C8	122.30 (13)
C3—C2—C1	116.76 (14)	C14—C9—C8	117.79 (13)
C3—C2—H2	121.6	C11—C10—C9	119.64 (17)
C1—C2—H2	121.6	C11—C10—H10	120.2
C2—C3—C4	121.88 (15)	C9—C10—H10	120.2
C2—C3—H3	119.1	C12—C11—C10	119.92 (19)
C4—C3—H3	119.1	C12—C11—H11	120.0
C5—C4—C3	121.72 (15)	C10—C11—H11	120.0
C5—C4—H4	119.1	C13—C12—C11	120.55 (18)
C3—C4—H4	119.1	C13—C12—H12	119.7
C4—C5—C6	117.55 (14)	C11—C12—H12	119.7
C4—C5—H5	121.2	C12—C13—C14	120.36 (19)
C6—C5—H5	121.2	C12—C13—H13	119.8
N2—C6—C5	129.77 (12)	C14—C13—H13	119.8
N2—C6—C1	109.86 (11)	C13—C14—C9	119.56 (17)
C5—C6—C1	120.36 (12)	C13—C14—H14	120.2
N2—C7—N1	112.86 (11)	C9—C14—H14	120.2
			
C7—N1—C1—C2	179.42 (14)	C1—N1—C7—C8	176.10 (11)
C7—N1—C1—C6	−0.39 (14)	N2—C7—C8—O1	160.44 (13)
N1—C1—C2—C3	−179.47 (15)	N1—C7—C8—O1	−14.80 (19)
C6—C1—C2—C3	0.3 (2)	N2—C7—C8—C9	−17.95 (19)
C1—C2—C3—C4	0.2 (2)	N1—C7—C8—C9	166.81 (11)
C2—C3—C4—C5	−0.6 (3)	O1—C8—C9—C10	142.64 (14)
C3—C4—C5—C6	0.5 (3)	C7—C8—C9—C10	−39.03 (18)
C7—N2—C6—C5	−179.18 (15)	O1—C8—C9—C14	−35.36 (19)
C7—N2—C6—C1	−0.19 (14)	C7—C8—C9—C14	142.97 (12)
C4—C5—C6—N2	178.85 (14)	C14—C9—C10—C11	0.0 (2)
C4—C5—C6—C1	0.0 (2)	C8—C9—C10—C11	−177.95 (14)
N1—C1—C6—N2	0.37 (14)	C9—C10—C11—C12	−2.2 (3)
C2—C1—C6—N2	−179.47 (12)	C10—C11—C12—C13	2.0 (3)
N1—C1—C6—C5	179.46 (13)	C11—C12—C13—C14	0.6 (3)
C2—C1—C6—C5	−0.4 (2)	C12—C13—C14—C9	−2.8 (3)
C6—N2—C7—N1	−0.06 (14)	C10—C9—C14—C13	2.5 (2)
C6—N2—C7—C8	−175.65 (11)	C8—C9—C14—C13	−179.45 (14)
C1—N1—C7—N2	0.29 (15)		

### Hydrogen-bond geometry (Å, °)

**Table:**

*D*—H···*A*	*D*—H	H···*A*	*D*···*A*	*D*—H···*A*
N1—H1···N2^i^	0.90 (2)	1.95 (2)	2.829 (2)	164 (1)

Symmetry codes: (i) −*x*+1/2, *y*+1/2, *z*.
